# Antibacterial Composites of Cuprous Oxide Nanoparticles and Polyethylene

**DOI:** 10.3390/ijms20020439

**Published:** 2019-01-21

**Authors:** Yanna Gurianov, Faina Nakonechny, Yael Albo, Marina Nisnevitch

**Affiliations:** Department of Chemical Engineering, Biotechnology and Materials, Ariel University, Kyriat-ha-Mada, Ariel 4070000, Israel; yannag@ariel.ac.il (Y.G.); fainan@ariel.ac.il (F.N.); yaelyt@ariel.ac.il (Y.A.)

**Keywords:** cuprous oxide nanoparticles, linear low-density polyethylene, composites, adhesives, antibacterial activity, water disinfection

## Abstract

Cuprous oxide nanoparticles (Cu_2_ONPs) were used for preparing composites with linear low-density polyethylene (LLDPE) by co-extrusion, thermal adhesion, and attachment using ethyl cyanoacrylate, trimethoxyvinylsilane, and epoxy resin. The composites were examined by Scanning electron microscope and tested for their antibacterial activity against Gram-positive *Staphylococcus aureus* and Gram-negative *Escherichia coli*. All of these composites—except for the one obtained by extrusion—eradicated cells of both bacteria within half an hour. The composite prepared by thermal adhesion of Cu_2_ONPs on LLDPE had the highest external exposure of nanoparticles and exhibited the highest activity against the bacteria. This composite and the one obtained using ethyl cyanoacrylate showed no leaching of copper ions into the aqueous phase. Copper ion leaching from composites prepared with trimethoxyvinylsilane and epoxy resin was very low. The antibacterial activity of the composites can be rated as follows: obtained by thermal adhesion > obtained using ethyl cyanoacrylate > obtained using trimethoxyvinylsilane > obtained using epoxy resin > obtained by extrusion. The composites with the highest activity are potential materials for tap water and wastewater disinfection.

## 1. Introduction

Polymeric materials have long become an integral part of our lives. They are used in most industrial fields, including the textile industry, food packaging, medical device production, and water supply and purification systems [1]. In the latter, piping for water transportation is made from polyolefins, such as polyvinylchloride [2], different kinds of polyethylene [3], polypropylene, and polybutylene [4,5]. Polyolefins do not possess antibacterial properties, and this is the cause for various problems that accompany water transportation, such as contamination by microorganisms, biofilm formation [6], and adherence of fungi and viruses to pipe walls. These problems may lead to the propagation of serious infections among humans. According to the World Health Organization (WHO), waterborne diseases lead to the deaths of 3.4 million people annually, most of whom are children, as a result of inaccessibility to clean water [7]. It is thus necessary to carry out effective water disinfection in order to combat and prevent water contamination.

The most common treatment for water purification from harmful microorganisms is chlorination. This treatment has been demonstrated to be efficacious for destroying microorganisms. However, it has several disadvantages, including the production of toxic, mutagenic, and carcinogenic disinfection byproducts [8,9].

It is possible to replace conventional water treatment methods by methods that are as effective but less toxic in order to improve the drinking water quality. One alternative method is to treat water in plumbing systems by antibacterial materials such as embedding heavy metals into the pipelines, thus eradicating pathogens before they reach the tap faucet. The most widely used heavy metals with proven efficacy against various microorganisms are silver, copper, and zinc [10]. These metals have been applied for years as antibacterial agents in industries, healthcare institutions, and agriculture [11]. Moreover, copper also possesses antiviral [12] and antifungal [13,14] properties. The exact mechanism of the antimicrobial action of these metals is still not totally clear due to many factors. The main suggested mechanism of copper activity against pathogens relates to the ability of copper ions to penetrate through the bacterial cell wall or outer membrane and bind to DNA, thus blocking the cell replication process [15]. In addition, high concentrations of copper ions stimulate oxidative stress, such as the generation of reactive oxygen species (ROS) [16,17,18], lipid peroxidation, [19,20] and protein oxidation [21].

Several studies show that copper nanoparticles (CuNPs) are more effective antibacterial agents than the same quantity of copper microparticles [22]. Copper oxide nanoparticles (NPs) also exhibit strong antibacterial activity by suppressing bacterial cell growth [23], and this activity even exceeds that of the metal copper NPs [24]. Both copper oxides (CuO and Cu_2_O) embedded into polyvinyl chloride (PVC) demonstrated high ability to inhibit bacterial adhesion to PVC when tested against *Escherichia coli* (*E. coli*) cells. It should be mentioned that Cu_2_O-PVC composites were more effective than CuO-PVC in preventing *E. coli* biofilm formation [25].

There are two approaches for the production of metal/polymer composites using NPs—in situ, where polymer matrices serve as reaction media for NP synthesis, and ex situ, where NPs are obtained beforehand and are later incorporated into a polymer [11]. The latter approach enables more precise dosing and distribution of NPs in polymers.

The aim of the present study is to propose simple approaches for immobilization of cuprous oxide nanoparticles (Cu_2_ONPs) for preparing composites with polyethylene that exhibit antibacterial properties for water disinfection.

## 2. Results and Discussion

### 2.1. Immobilization of Cu_2_ONPs onto A Solid Phase

Domestic water piping is made of polymers. Linear low density polyethylene (LLDPE) was therefore chosen as a support for immobilization of Cu_2_ONPs. The use of LLDPE enabled application of various approaches for immobilization of Cu_2_ONPs and easy handling of the prepared composites. Several methods were used for NP immobilization.

**Figure 1:**
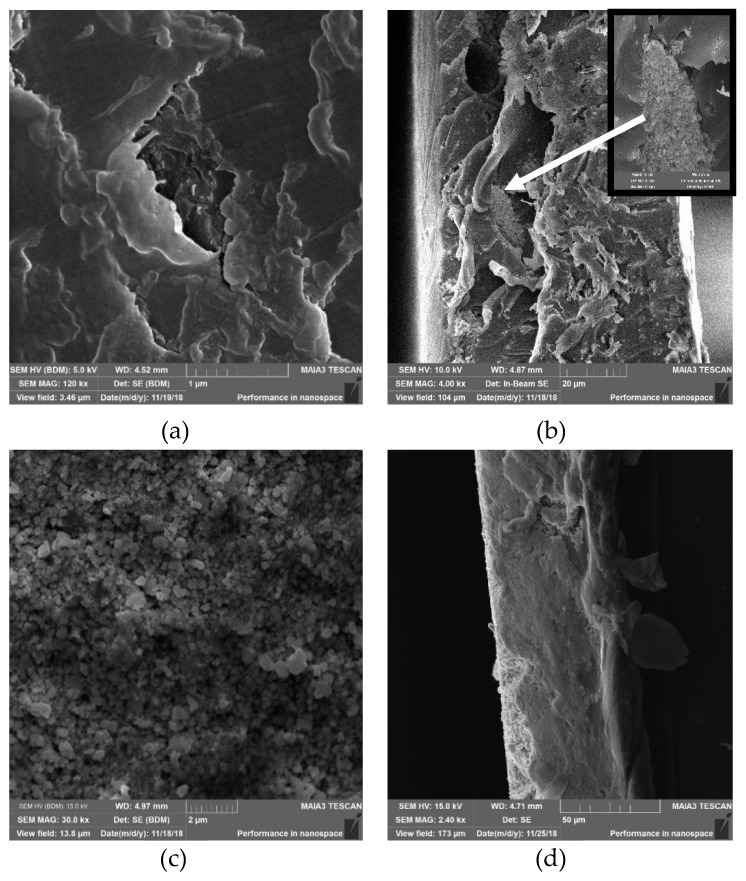

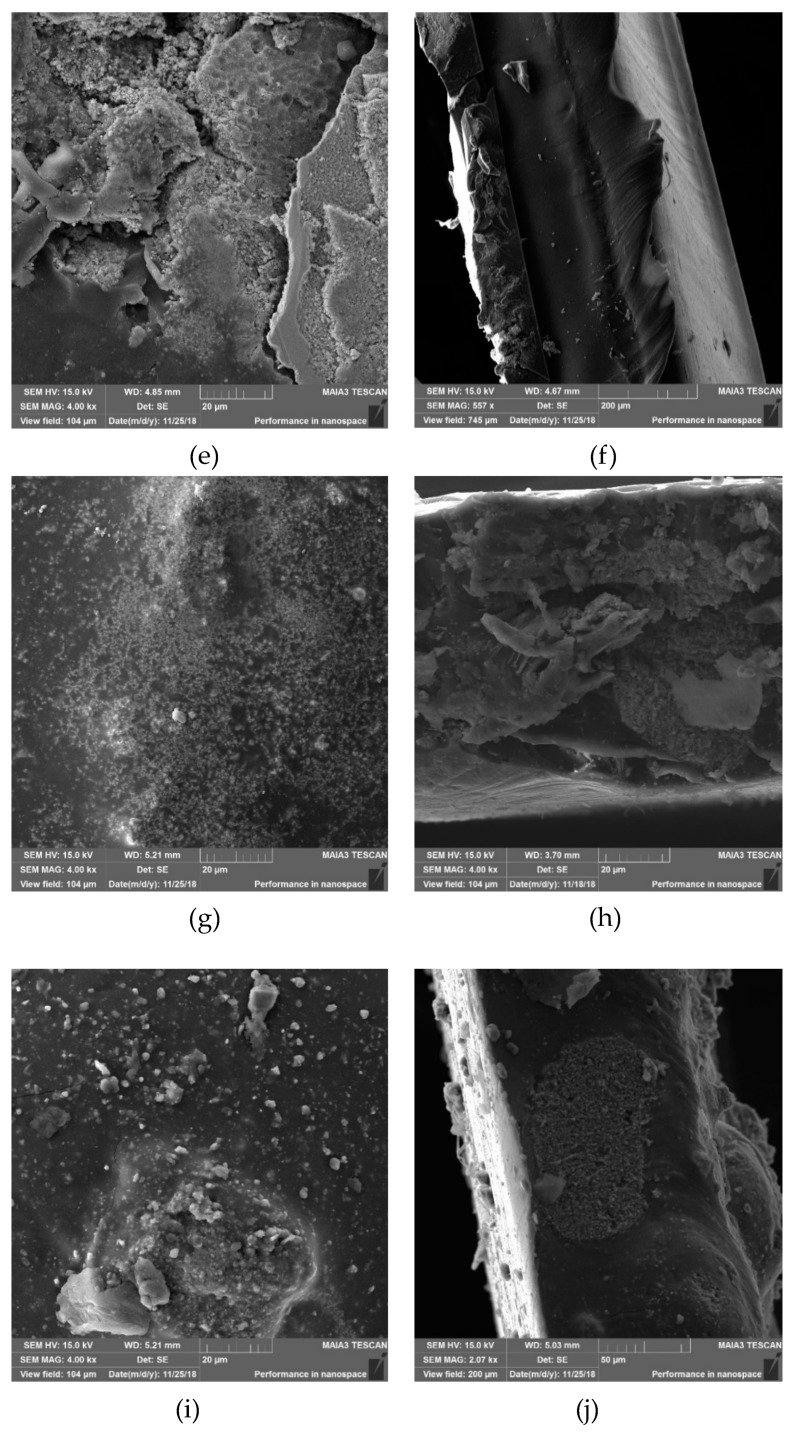
Scanning electron microscope (SEM) micrographs of Cu_2_ONPs immobilized onto linear low-density polyethylene (LLDPE) by extrusion: (**a**) surface and (**b**) cross-section images; by thermal adhesion: (**c**) surface and (**d**) cross-section images; using ethyl cyanoacrylate: (**e**) surface and (**f**) cross-section images; using epoxy resin: (**g**) surface and (**h**) cross-section images; using trimethoxyvinylsilane: (**i**) surface and (**j**) cross-section images.

The first method was based on extrusion of a mixture of LLDPE beads with Cu_2_ONPs. As a result, polymeric strips with Cu_2_O impregnated into the entire volume of polyethylene (PE) were obtained. The strips were evenly colored in brown-red, which is characteristic for Cu_2_ONPs, and seemed to have a homogeneous distribution of NPs. However, Scanning electron microscope (SEM) examination of the strips showed that their surface was composed mostly of PE (Figure 1a), while inside the strips, rare clusters of Cu_2_ONPs were surrounded by massive polymeric parts, as can be seen in the cross-section of the strip (Figure 1b and the inset in Figure 1b). It can be assumed that practically no NPs were exposed on the surface of the strips and that the Cu_2_ONPs were distributed very unevenly inside the polymer.

The second method of immobilization was thermal adhesion of Cu_2_ONPs distributed onto a surface of the heated, melting LLDPE and pressed into the melted polymer under slight pressure. This approach led to results that were different from those obtained by the extrusion method. Figure 1c shows that after this treatment, the polymeric surface was covered with clearly distinguishable Cu_2_ONPs (Figure 1c), and imaging of the polymer cross-section indicated a distinct two-layer structure of the obtained composite where the external layer composed of Cu_2_ONPs had a thickness of 72.7 ± 0.3 µm (Figure 1d).

Other immobilization methods were based on attaching Cu_2_ONPs onto the LLDPE surface using three types of adhesives: ethyl cyanoacrylate, epoxy resin, and trimethoxyvinylsilane. In the case of the ethyl cyanoacrylate, the attached NPs were distributed over the entire polymeric surface and were partially exposed on the external side (Figure 1e). The layer of the attached NPs was quite even and had a thickness of 97.2 ± 2.2 µm (Figure 1f). Epoxy resin yielded a rather even distribution of Cu_2_ONPs on the polymer surface, but the NPs were mostly covered by a film of the adhesive (Figure 1g). The layer thickness in this case was 89.7 ± 0.2 µm (Figure 1h). Cu_2_ONPs attachment using trimethoxyvinylsilane yielded an uneven surface distribution of the NPs, which were mostly covered by an adhesive film. However, in some areas, good exposure of NPs was clearly evident (Figure 1i). The adhesive layer containing NPs had a thickness of 152.1 ± 1.5 µm (Figure 1j).

**Figure 2:**
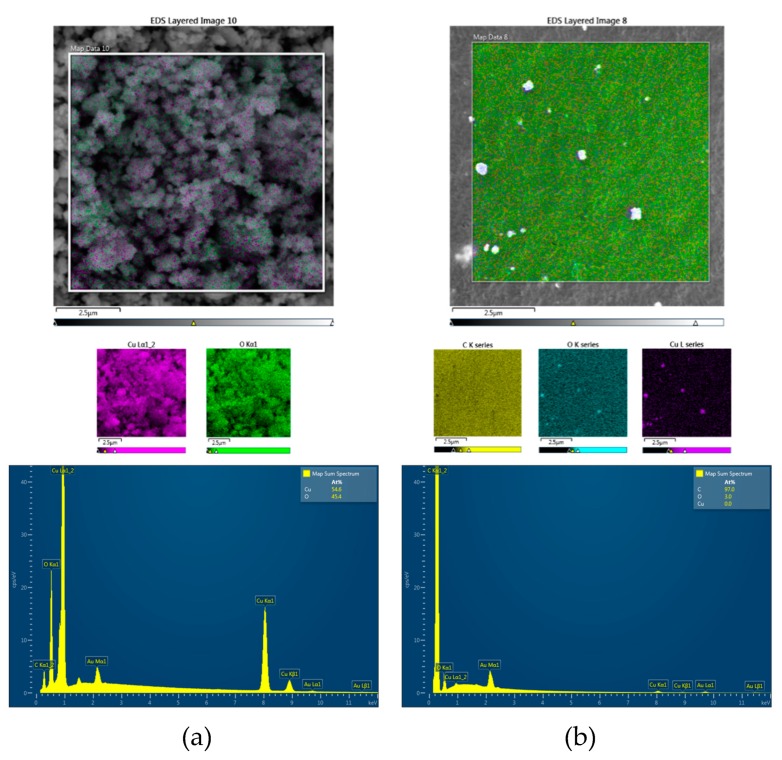

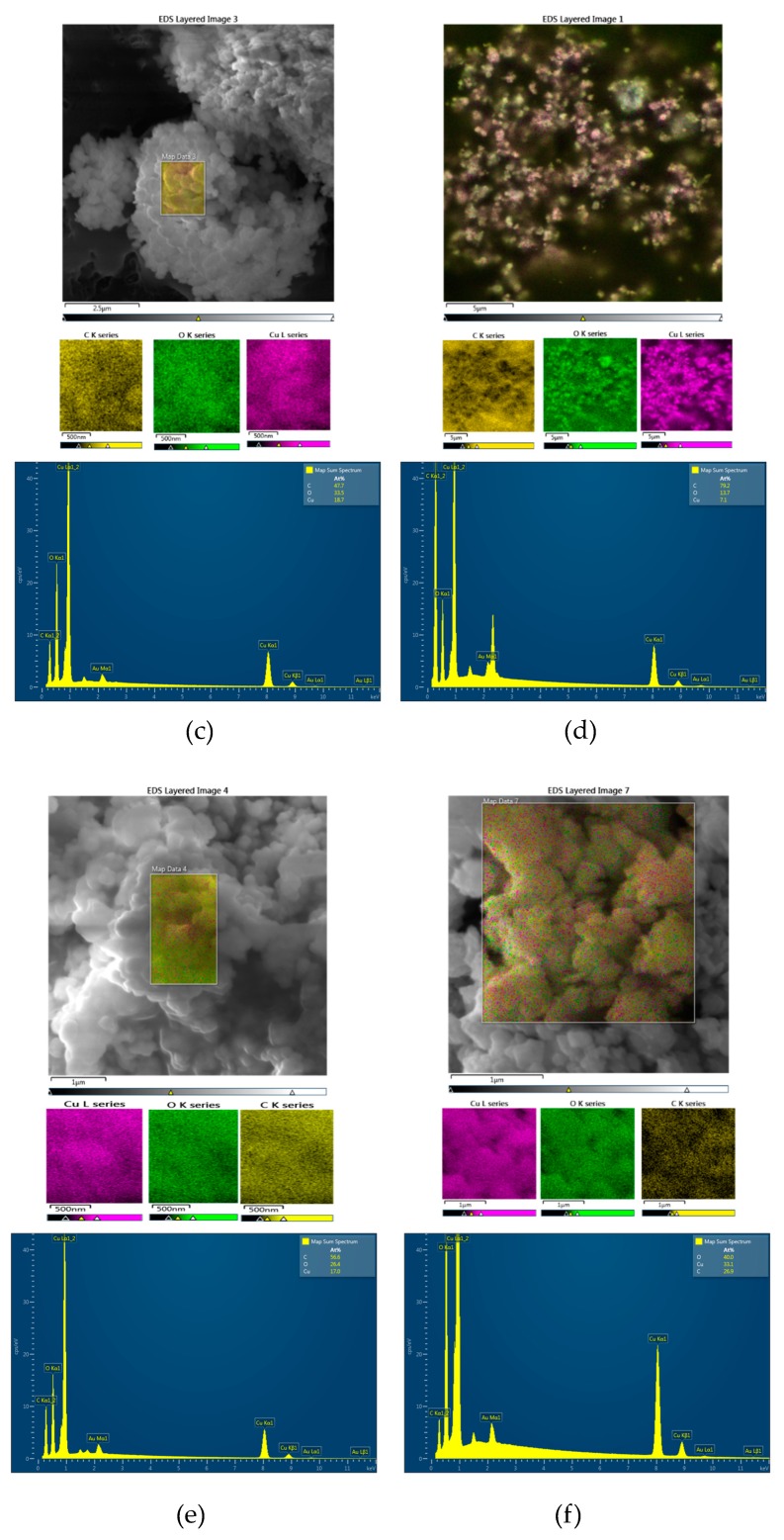
Surface elemental mapping of gold-coated (**a**) powder Cu_2_ONPs; (**b**) Cu_2_ONPs immobilized onto LLDPE by extrusion; (**c**) Cu_2_ONPs immobilized onto LLDPE using ethyl cyanoacrylate; (**d**) Cu_2_ONPs immobilized onto LLDPE using epoxy resin; (**e**) Cu_2_ONPs immobilized onto LLDPE using trimethoxyvinylsilane; (**f**) Cu_2_ONPs immobilized onto LLDPE by thermal adhesion. Each sample is characterized by an energy dispersive X-ray spectroscopy (EDS) layered image (upper panel) with individual elemental mapping showing oxygen as green, copper as purple, and carbon as yellow (middle panel), and EDS spectrum (bottom panel).

**Figure 3:**
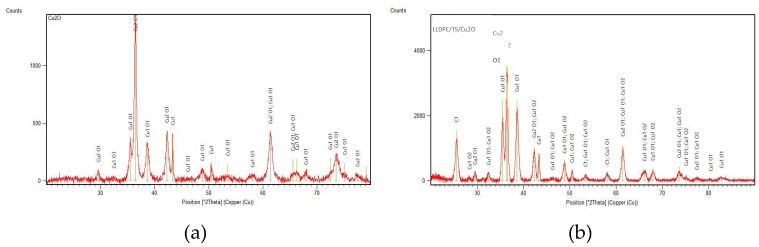
X-ray diffraction (XRD) analysis of (**a**) powder Cu_2_ONPs and (**b**) immobilized using trimethoxyvinylsilane Cu_2_ONPs (LLDPE/TS/Cu_2_O).

The surfaces of all the composites were characterized by elemental mapping. Figure 2 shows that unbound Cu_2_ONPs exposed only copper and oxygen atoms (Figure 2a), whereas all composites showed the presence of carbon, copper, and oxygen, as expected (Figure 2b–e). In the composite prepared by extrusion (Figure 2b), practically no copper and oxygen atoms were found on the surface. This supports our previous observation that Cu_2_ONPs are located inside the polymer and are not exhibited on the surface of the composite (Figure 1a). For the rest of the composites, the molar fraction of oxygen was higher than expected for Cu_2_O, which can be explained by the presence of oxygen atoms in the adhesives, and probably also by partial oxidation of Cu^+1^ to Cu^+2^ in the external layer of the NPs, which was exposed to air. X-ray diffraction (XRD) analysis of the composite obtained using trimethoxyvinylsilane showed the presence of three copper species—Cu^0^, Cu^+1,^ and Cu^+2^ (Figure 3)—where Cu^+1^ was the prevalent form for both powder Cu_2_ONPs (Figure 3a) and immobilized Cu_2_ONPs (Figure 3b). We therefore concluded that the bulk of Cu_2_ONPs retain their chemical structure.

### 2.2. Leaching of Copper from Immobilized Cu_2_ONPs

Copper ions are well known for their antibacterial activity. However, they are toxic to humans (even at low concentrations), causing gastrointestinal distress and liver or kidney damage [26,27]. Standards for copper ions in tap water are therefore very strict, and the maximum permitted copper concentration in drinking water is 1.4–2 ppm [28,29]. The copper ion concentration cannot exceed 2–15 ppm in sanitary and combined waste discharges [30,31].

Since the probability of copper leaching into water from the Cu_2_ONPs-polymer composites in the case of immobilization by surface attachment seemed to be high, we tested the copper ion concentration of the composite samples after immersing them in tap water for one month under the following conditions: temperature 20.1 ± 1.0 °C, pH 8.2 ± 0.1, dissolved oxygen concentration 5.4 ± 0.3 mg/L, and salinity 0.301 ± 0.034 mS.

**Figure 4:**
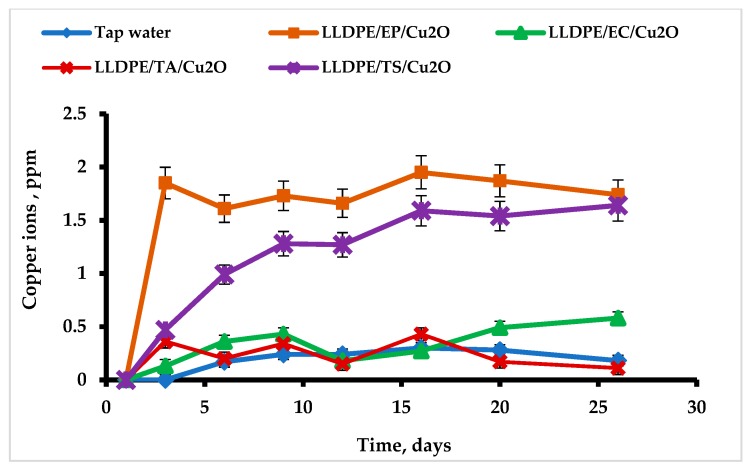
Testing the leaching of copper ions into tap water from LLDPE with Cu_2_ONPs attached to the surface using ethyl cyanoacrylate (EC), trimethoxyvinylsilane (TS), epoxy resin (EP), and thermal adhesion (TA). Control—tap water.

The results of this test are presented in Figure 4. The copper concentration in the control tap water was 0.24 ± 0.02 ppm. Thus, it did not exceed the maximum permitted concentration. No leaching of copper ions into the aqueous phase was detected when Cu_2_ONPs were immobilized with ethyl cyanoacrylate and thermal adhesion, and the average copper concentration was 0.35 ± 0.06 ppm and 0.25 ± 0.05 ppm, respectively. These results were similar to the tap water control series, with a *P*-value of 0.15 for ethyl cyanoacrylate and 0.48 for thermal adhesion. The picture was quite different for the two other adhesives where the copper concentration in water increased with time (Figure 4).

In both cases, the copper leaching curve could be described as a saturation curve; for the composite produced with epoxy resin, saturation at ca. 1.9 ppm was already reached after three days, and for the trimethoxyvinylsilane-based composite, saturation at ca. 1.6 ppm was reached after 16 days. These values were significantly different from the tap water. For the former composite, the *P*-value was 0.0032, and for the latter, it was 0.010. Small amounts of Cu_2_ONPs were probably not attached well enough to the polymer support and leached upon contact with water. It should be emphasized that the experiment was performed in a batch mode, i.e., the leached copper ions accumulated in the aqueous phase during the experiment. In a continuous regime, the concentration of the leached copper is considered to be lower, since the leached ions will be removed by the water flow.

Average rates of copper leaching from the composites prepared by various methods and total amount of leached copper are presented in Table 1. It can be seen that the rate of leaching and the percent of leached copper were very low in the case of the composites prepared by thermal attachment and using ethyl cyanoacrylate. In the case of the two other methods, these parameters were higher but leached copper still comprised only ca. 1% from the applied amount. These findings indicate that copper leaching can be regarded as being either negligible or very low in all cases.

### 2.3. Antibacterial Activity of Immobilized Cu_2_ONPs

**Figure 5:**
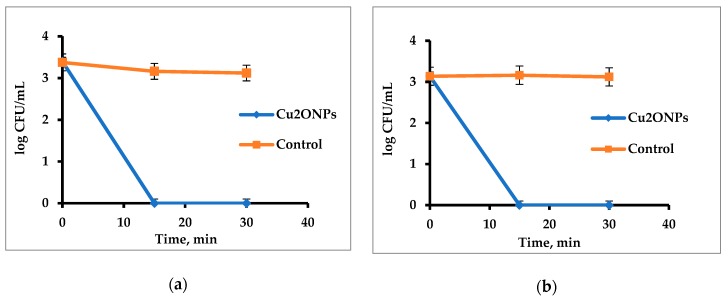
Activity of free Cu_2_ONPs against (**a**) *Staphylococcus aureus* and (**b**) *Escherichia coli* cells. Control—untreated bacterial cells.

Firstly, antibacterial activity of free suspended Cu_2_ONPs was tested against Gram-positive *Staphylococcus aureus* (*S. aureus*) and Gram-negative *E. coli*. Figure 5 shows that the NPs were very active and eradicated the cells of both bacteria after 15 min.

After that, we studied the ability of immobilized Cu_2_ONPs to eradicate the same bacteria. For this purpose, the samples of LLDPE bearing Cu_2_ONPs were placed into bacterial suspensions with known concentrations and incubated for half an hour. After short washing, the polymeric samples were then transferred into a dish with a new portion of fresh suspension with the same initial concentration. This procedure was repeated several times. Each time, the bacterial concentration was analyzed before and after the incubation. In the control series, bacteria were incubated in the absence of any polymer and in the presence of samples of LLDPE and LLDPE coated with adhesives without the addition of Cu_2_ONPs.

**Figure 6:**
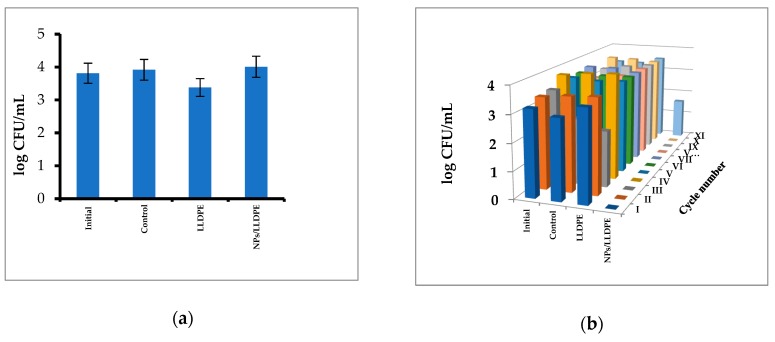

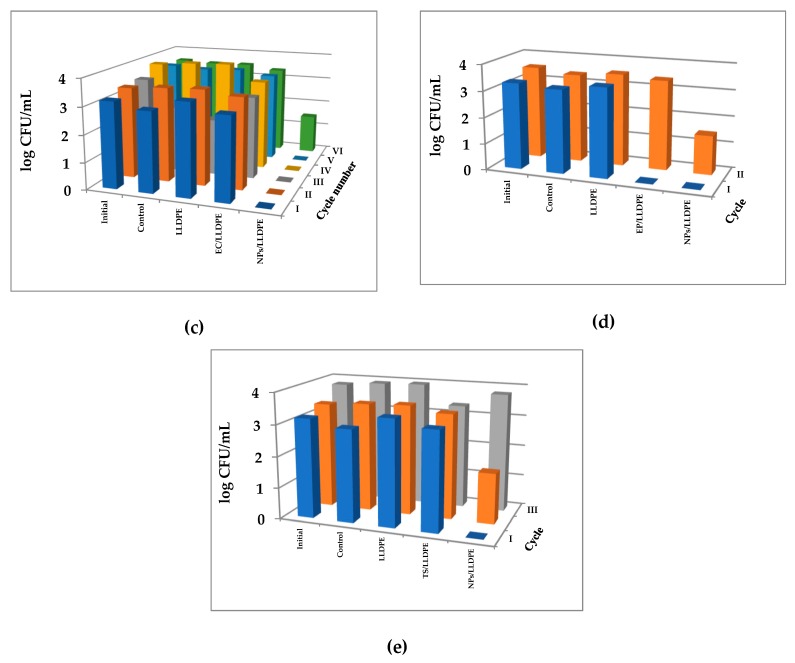
Activity of the Cu_2_ONPs-LLDPE composites against *S. aureus* cells of composites obtained by: (**a**) extrusion; (**b**) thermal adhesion; (**c**) using ethyl cyanoacrylate; (**d**) using epoxy resin; (**e**) using trimethoxyvinylsilane. Roman figures show the number of re-use cycles. Initial—*S. aureus* cells before the incubation, control—*S. aureus* cells after 30 min incubation, LLDPE—*S. aureus* cells after 30 min incubation with LLDPE, EC/LLDPE—*S. aureus* cells after 30 min incubation with LLDPE coated with an ethyl cyanoacrylate layer, TS/LLDPE—*S. aureus* cells after 30 min incubation with LLDPE coated with a trimethoxyvinylsilane layer, EP/LLDPE—*S. aureus* cells after 30 min incubation with LLDPE coated with an epoxy resin layer, NPs/LLDPE—*S. aureus* cells after 30 min incubation with the Cu_2_ONPs-LLDPE composite. In all cases, relative standard errors did not exceed 10%.

The results of testing the activity of immobilized Cu_2_ONPs against *S. aureus* are presented in Figure 6. It can be seen that Cu_2_ONPs immobilized by extrusion did not exhibit any toxicity against the cells (Figure 6a), whereas all other LLDPE samples bearing Cu_2_ONPs on the surface were active against *S. aureus* and totally eradicated the cells within half an hour (Figure 6b–e).

The highest activity was demonstrated by the sample obtained by thermal adhesion of Cu_2_ONPs onto the LLDPE surface. This sample demonstrated an ability to kill the cells over the course of 10 cycles of re-use (Figure 6b). Other composites were less active against *S. aureus*; Cu_2_ONPs attached to the LLDPE surface by ethyl cyanoacrylate totally destroyed the cells over the course of five cycles of re-use and decreased the cell concentration by 1.5–2 log_10_ during cycles six through 10 (Figure 6c). The Cu_2_ONPs-LLDPE composites obtained with epoxy resin and trimethoxyvinylsilane were active only for a single use, caused only a 2 log_10_ decrease in the *S. aureus* concentration during the second re-use, and were inactive during the third re-use (Figure 6d,e, respectively).

No decrease in the *S. aureus* concentration was observed in the control experiments in the absence of the composites and in the presence of uncoated LLDPE or LLDPE coated with each of the adhesives (Figure 6) except the LLDPE coated with epoxy resin, which was toxic for bacteria in the first cycle (Figure 6d).

**Figure 7:**
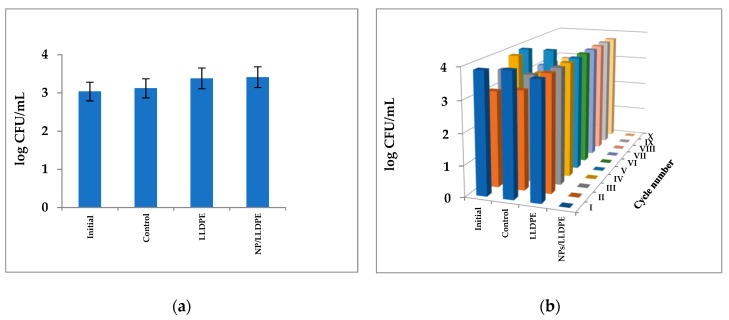

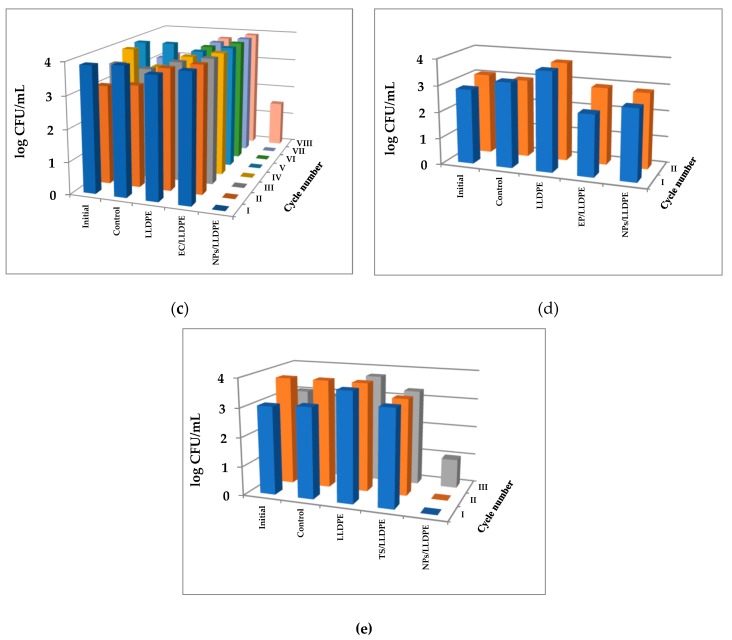
Activity of the Cu_2_ONPs-LLDPE composites against *E. coli* cells of composites obtained by: (**a**) extrusion; (**b**) thermal adhesion; (**c**) using ethyl cyanoacrylate; (**d**) using epoxy resin (**e**) using trimethoxyvinylsilane. Roman figures show the number of re-use cycles. Initial—*E. coli* cells before the incubation, control—*E. coli* cells after 30 min incubation, LLDPE—*E. coli* cells after 30 min incubation with LLDPE, EC/LLDPE—*E. coli* cells after 30 min incubation with LLDPE coated with an ethyl cyanoacrylate layer, TS/LLDPE—*E. coli* cells after 30 min incubation with LLDPE coated with a trimethoxyvinylsilane layer, EP/LLDPE—*E. coli* cells after 30 min incubation with LLDPE coated with an epoxy resin layer, NPs/LLDPE—*E. coli* cells after 30 min incubation with the Cu_2_ONPs-LLDPE composite. In all cases, relative standard errors did not exceed 10%.

The results of the experiments of testing the antibacterial activity of the composites against Gram-negative *E. coli* were very similar to those found for *S. aureus*. The Cu_2_ONPs-LLDPE composite obtained by extrusion was also inactive against *E. coli* (Figure 7a). The composite obtained by thermal adhesion exhibited the highest activity, which was retained for ten cycles of re-use (Figure 7b). The composite obtained using ethyl cyanoacrylate was active for seven re-use cycles, and in the eighth cycle, the concentration of *E. coli* decreased by 2.5 log_10_ (Figure 7c). Contrary to *S. aureus*, the composite produced with epoxy resin was inactive—even in the first use (Figure 7d). The composite obtained using trimethoxyvinylsilane was active against *E. coli* during two cycles of use, but in the third re-use, the cell concentration dropped by 2 log_10_ only (Figure 7e). No decrease in *E. coli* cell concentration was registered in the control experiments (Figure 7).

Taking the results of Gram-positive and Gram-negative bacteria eradication into account, the overall antibacterial activity of the obtained composites can be rated as follows: obtained by thermal adhesion > attached using ethyl cyanoacrylate > attached using trimethoxyvinylsilane > attached using epoxy resin > obtained by extrusion. The different antimicrobial activities of the obtained composites can be explained by different exposure rates of Cu_2_ONPs to the aqueous phase, as demonstrated in Figure 1, and by different degrees of leaching of copper ions from the polymer surface. The Cu_2_ONPs were highly exposed in the case of thermal adhesion to the polymer, and in this case, copper leaching was zero. This composite also demonstrated the highest activity against the bacterial cells. In the case of ethyl cyanoacrylate, the rate of Cu_2_ONPs exposure was less than in the former case but still high enough, and no leaching of copper ions was observed. These features explain the second position of this composite in the antibacterial rating. The composites produced using trimethoxyvinylsilane and epoxy resin had a very low rate of Cu_2_ONPs exposure and showed leaching of copper ions into the water. Thus, contact between bacterial cells and immobilized Cu_2_ONPs was very poor and probably even decreased with time since copper leached into the aqueous phase.

The high antibacterial activity of the composites obtained by thermal adhesion and using the ethyl cyanoacrylate makes them potential materials for application in tap or waste water disinfection in batch or continuous regimes. Since these composites showed no leaching of copper ions into the aqueous phase (Figure 4), it can be concluded that cell killing occurs upon direct contact between the cells and the Cu_2_ONPs found on the surface. The absence of copper ions in the aqueous environment supports this conclusion.

## 3. Materials and Methods

### 3.1. Materials

The LLDPE was purchased from Sigma-Aldrich, Israel Ltd. Cu_2_ONPs of 18 nm size were purchased from US Research Materials (Houston, TX, USA). Ethyl cyanoacrylate (“super glue”) was purchased from Loctite^®^ (Westlake, OH, USA), trimethoxyvinylsilane (Hybrifix Super 7) was purchased from Den Braven Sealants B.V (Oosterhout, Netherlands), and epoxy resin and polyamine hardener were purchased from Evobond^®^ (Kaohsiung City, Taiwan).

### 3.2. Immobilization of Cu_2_ONPs onto LLDPE Polymer by Thermal Adhesion

Three grams of LLDPE pellets were melted at 130 °C on a Kapton polyimide film (Shagal Marketing Solutions Ltd., Modiin, Israel). The melted polymer was coated with another polyimide film and a thick stainless steel plate and pressed under moderate pressure to obtain a 1 mm thick layer. After removal of the plate and the upper polyimide film, 0.15 g of Cu_2_ONPs were dispersed on the molten polymer using a sieve, covered with another polyimide film and the plate and slightly pressed in. The samples were cooled to room temperature.

### 3.3. Attachment of Cu_2_ONPs to the LLDPE Polymer

The LLDPE sample was prepared as described in Section 3.2. After cooling the sample to room temperature, a layer of one of the adhesives (ethyl cyanoacrylate, trimethoxyvinylsilane, or epoxy resin) was applied to the polymer by a draw down technique, and 0.15 g of cuprous oxide NPs were dispersed on the adhesives immediately using a sieve. The samples were air-dried for 24 h under sterile conditions.

### 3.4. Immobilization of Cu_2_ONPs into A LLDPE Matrix by Extrusion

Immobilization of the Cu_2_ONPs into LLDPE was performed by a co-extrusion technique using an extruder (Allspeeds Ltd., Accrington, England) under an inlet temperature of 170 °C and an outlet temperature of 210 °C. For this purpose, a mixture of 50 g of polymer beads and 2.5 g of Cu_2_ONPs was placed in a feed, and the extruder was activated to melt the mixture at 30 rpm. The resulting fluid composition was pushed through a die with a flat 1 × 19.6 mm section. This procedure yielded polymeric rods with the incorporated Cu_2_ONPs. The rods were chopped into 5 cm-long pieces.

### 3.5. Bacterial Growth

Inoculums of Gram-positive *S. aureus* (ATCC 25923) and Gram-negative *E. coli* (ATCC 10798) were grown in brain heart infusion broth (BH; Acumedia, Lansing, MI, USA) and Luria Bertani broth (LB; Acumedia, Lansing, MI, USA), respectively, at 37 ± 1°C under shaking at 150 rpm for 24 h, diluted 1:100 with the corresponding medium, and incubated again for ca. 2 h at 37 ± 1°C until reaching OD_660nm_ = 0.1. The bacterial suspensions were diluted with sterile saline to a final concentration of 10^3^ or 10^4^ CFU/mL.

### 3.6. Testing of Antibacterial Activity

The antibacterial activity of free and immobilized Cu_2_ONPs samples was tested against *S. aureus* and *E. coli*; 0.15 g of Cu_2_ONPs (powder) or 3 g of Cu_2_ONPs-LLDPE composites were added to 20 mL of bacterial culture and incubated at 37 ± 1°C by shaking at 120 rpm for 30 min. The samples were then diluted by one and two decimal dilutions and 100 µL of these samples were distributed onto BH or LB agar plates in the cases of *S. aureus* and *E. coli*, respectively. The plates were incubated overnight at 37 ± 1 °C, and the bacterial colony forming units (CFU) were counted using a colony counter Scan 500 (Interscience, Saint Nom la Bretèche, France). The bacterial concentration was determined while taking the appropriate dilutions into account.

### 3.7. Leaching of Copper Ions from Samples of Immobilized Cu_2_ONPs into Tap Water

Six grams of LLDPE with 0.3 g of immobilized Cu_2_ONPs were added to 100 mL of tap water and stirred at 120 rpm with a magnetic stirrer for one month under sampling twice a week. One mL samples were diluted by 9 mL of distilled water and filtered through Polyvinylidene fluoride (PVDF) filters with 0.45 µm pore size (Membrane Solutions, Kent, WA, USA). The copper ion concentration in the samples was measured using an ICP-AES (Spectro Arcos, Ametek^®^, Berwyn, PA, USA) instrument. The pH and temperature were tested with a HI 2211 pH/OPR meter (HANNA Instruments, Woonsocket, RI, USA). Dissolved oxygen concentration was measured by a DO-5510 oxygen meter (Lutron, Taiwan). Salinity was measured with a CD-4303HA conductivity meter (Lutron, Taiwan).

### 3.8. SEM Imaging and EDS Analysis of Immobilized Cu_2_ONPs

Imaging of surfaces and cross-sections of immobilized Cu_2_ONPs was performed with an SEM microscope (Tescan MAIA3, Triglav™, Brno, Czech Republic). The samples were placed onto a carbon tape and covered with a 10 nm gold layer using a Q150T ES Quorum coater (Quorum Technologies Ltd., Lewes, UK) under a sputter current of 12 mA for 30 s. SEM measurements were performed at operating voltages of 5, 10, and 15 kV and at magnifications of ×557, ×2.07 k, ×2.40 k, ×4.00 k, ×30.0 k, and ×120 k. The samples were detected with In-beam SE (secondary electrons) and SE-BDM (beam deceleration mode) detectors. Elemental analysis of the samples was performed by energy dispersive X-ray analysis in SEM mode under the resolution of 127 eV using a X-Max^N^ SDD detector 51-xmx1010 (Oxford Instruments NanoAnalysis, High Wycombe, UK).

### 3.9. XRD Analysis of Powder and Immobilized Cu_2_ONPs

Phase analysis of the samples was carried out using a Panalytical X’Pert Pro X-ray powder diffractometer (Malvern Panalytical Ltd., Malvern, UK) with Cu Kα radiation (λ = 0.154 nm) for phase identification. Full pattern identification was performed by the X’Pert HighScore Plus software package, version 2.2e (2.2.5) (Malvern Panalytical Ltd., Malvern, UK). XRD patterns were obtained at 40 kV and 40 mA. For immobilized Cu_2_ONPs, the grazing incidence geometry with an incident angle of −5° was applied. The XRD patterns were recorded in the 2θ range of 20–80° with a step size of 0.02° and time per step of 1 s. For powder Cu_2_ONPs, Bragg-Brentano geometry was applied. The XRD patterns were recorded in the 2θ range of 20–80° with a step size of 0.03° and time per step of 2 s.

### 3.10. Statistical Analysis

The results obtained from at least three independent experiments carried out in duplicates were analyzed by single-factor Analysis of Variance (ANOVA). The difference between the results was considered significant when the *P*-value was less than 0.05. Quantitative results are presented as the mean ± standard error.

## 4. Conclusions

Composites of cuprous oxide nanoparticles with linear low-density polyethylene showed no or very low leaching of copper ions into the aqueous phase and exhibited good antibacterial activity against *S. aureus* and *E. coli*.

## Figures and Tables

**Table 1:** Rate and amount of copper leaching from composites of Cu_2_ONPs and LLDPE.

Composite	Rate of Copper Leaching, µg cm^−2^ day^−1^	Percent of Leached Copper
Obtained by thermal adhesion	0.019	0.15
Obtained using ethyl cyanoacrylate	0.025	0.17
Obtained using epoxy resin	1.32	1.11
Obtained using trimethoxyvinylsilane	0.166	1.06

## Abbreviations

NPs Cu_2_ONPs PE	Nanoparticles Cuprous oxide nanoparticles Polyethylene
LLDPE EDS SEM XRD	Linear low-density polyethylene Energy-dispersive X-ray spectroscopy Scanning electron microscope X-ray diffraction
